# Unique primed status of microglia under the systemic autoimmune condition of lupus-prone mice

**DOI:** 10.1186/s13075-019-2067-8

**Published:** 2019-12-30

**Authors:** Atsushi Nomura, Daisuke Noto, Goh Murayama, Asako Chiba, Sachiko Miyake

**Affiliations:** 1 Department of Immunology, Juntendo University School of Medicine, 2-1-1 Hongo, Bunkyo-ku, Tokyo, 113-8421 Japan; 2 Department of Internal Medicine and Rheumatology, Juntendo University School of Medicine, Tokyo, Japan

**Keywords:** Primed microglia, Myeloid cells, Monocytes, Neurodegeneration, Autoimmunity

## Abstract

**Background:**

Systemic lupus erythematosus (SLE) is an autoimmune disease characterized by the production of various autoantibodies. This disease causes disabling neuropsychiatric symptoms even in the absence of apparent inflammation in the central nervous system (CNS), but the mechanisms involved remain unknown. Innate immune-mediated inflammation has attracted attention as a pathogenic mechanism in neuropsychiatric diseases.

**Methods:**

We investigated the CNS of lupus-prone mice focusing on innate immunity. Three strains of lupus-prone mice, FcγRIIB^−/−^*Yaa*, an F1 hybrid of NZB and NZW (NZB/NZW) mice, and MRL/Fas^lpr^ (MRL/lpr) mice were used to analyze CNS immunopathology.

**Results:**

Flow cytometry analysis demonstrated the numbers of brain CD45^+^ cells were increased compared with controls in lupus-prone mice. Upregulation of MHC class I and PDCA1 was observed in microglia and CD11b^+^ myeloid cells of lupus-prone mice, indicating they were activated in response to interferons (IFN). Microglial gene expression analysis of FcγRIIB^−/−^*Yaa* mice revealed the upregulation of IFN-responsive genes and inflammation-related genes including *Axl*, *Clec7a*, and *Itgax*, which were previously reported in neurodegenerative conditions and primed conditions. Upregulated chemokine gene expressions including *Ccl5* and *Cxcl10* were concurrent with increased numbers of T cells and monocytes, especially Ly6C^lo^ monocytes in the CNS. Upregulation of *Axl*, *Clec7a*, *Itgax*, *Ccl5*, and *Cxcl10* was also observed in NZB/NZW mice, indicating common lupus pathology. The primed status of microglia in FcγRIIB^−/−^*Yaa* mice was also demonstrated by morphological changes such as enlarged cell bodies with hypertrophic processes, and hyperreactivity to lipopolysaccharide. Immunohistochemistry of FcγRIIB^−/−^*Yaa* mice indicated reactive responses of astrocytes and vascular endothelium. Behavioral studies of FcγRIIB^−/−^*Yaa* mice revealed depressive-like behavior and heat hyperalgesia in the forced swim test and the tail-flick test, respectively.

**Conclusions:**

Our data indicated that microglia in lupus exhibit a unique primed phenotype characterized by the upregulated expressions of neurodegeneration-related genes and IFN-responsive genes. Interaction with peripheral cells and brain resident cells was presumed to orchestrate neuroinflammation. Targeting innate immune cells, such as microglia and monocytes, may be a promising therapeutic approach for neuropsychiatric SLE.

## Background

Systemic lupus erythematosus (SLE) is a systemic autoimmune disease characterized by the production of various autoantibodies and damage to multiple organs such as the kidneys, skin, and nervous system [1]. Neuropsychiatric systemic lupus erythematosus (NPSLE) includes neurologic syndromes of the central, peripheral, and autonomic nervous system as well as psychiatric syndromes and is classified into 19 syndromes according to the criteria of the American College of Rheumatology [2]. Neuropathy, seizures, and acute confusional states are attributed to autoantibodies and inflammatory mediators, and cerebrovascular diseases are caused by vascular events. Although symptoms such as mild forms of headache, cognitive impairment, and mood disorders are more frequently observed in SLE patients compared with control groups, they are sometimes considered not attributable to SLE [3], and their underlying mechanisms remain unclear. However, symptoms such as cognitive impairment and mood disorders in SLE patients are often accompanied by fatigue and pain [4], which are the principal symptoms of fibromyalgia and myalgic encephalomyelitis/chronic fatigue syndrome (ME/CFS). Even though these diseases do not exhibit clinical signs of systemic inflammation, inflammatory profiles were observed by detailed blood cytokine examinations [5, 6], and innate immune-mediated inflammation in the central nervous system (CNS) characterized by microglial activation detected by positron emission tomography (PET) has been reported in both diseases [7, 8].

Chronic neuroinflammation has attracted attention regarding its role in the pathogenesis of neurodegenerative and neuropsychiatric diseases. Microglia, resident hematopoietic myeloid cells in the CNS, play a central role in neuroinflammation. They are involved in the development and homeostasis of the brain as well as in acute and chronic inflammation [9]. Recent studies characterizing microglial phenotypes revealed that their polarizing status is diverse. In disorders such as Alzheimer’s disease, amyotrophic lateral sclerosis, multiple sclerosis, and aging, microglia have a unique inflammatory status related to neurodegeneration [10–12]. In these diseases, resident cells in the CNS, such as astrocytes and vascular endothelium, also contribute to the development of inflammation through their interactions with microglia and peripheral immune cells [13–15]. Furthermore, systemic inflammation is a well-known aggravating factor in neurodegenerative disorders such as Parkinson’s disease and Alzheimer’s disease [16, 17].

Lupus model mice were used to investigate the neuropsychiatric pathology of SLE and exhibited behavioral changes such as depression-like behavior, anxiety-like behavior, and cognitive dysfunction [18, 19]. Although the infiltration of lymphocytes into the choroid plexus and microglial activation resulting in damage to synapses have been reported in lupus-prone mice [20, 19], the pathological involvement of the CNS remains largely unknown. In the present study, we investigated immunological changes in the CNS of three strains of lupus-prone mice: FcγRIIB^−/−^*Yaa* mice, a lupus model created by the lack of FcγRIIB suppression and duplication of Toll-like receptor 7 (TLR7) by the *Yaa* gene [21]; an F1 hybrid between NZB and NZW (NZB/NZW) mice; and MRL/Fas^lpr^ (MRL/*lpr*) mice. In all three lupus-prone mouse strains, innate immune cells including myeloid cells and microglia were activated in the CNS. Changes in the gene expressions of microglia in FcγRIIB^−/−^*Yaa* mice were characterized by the upregulation of IFN-related genes and inflammation-related genes previously reported in neurodegenerative disorders. Morphological changes such as enlarged cell bodies, hypertrophic processes, and hyperreactivity to lipopolysaccharide (LPS) indicated their primed status [22]. We termed this microglial condition “lupus-associated microglia” (LAM), which represents the unique activation status of the CNS in lupus. This unique activation of microglia may provide a clue to understanding the disease pathology and developing therapeutic strategies for NPSLE.

## Materials and methods

### Mice

FcγRIIB^−/−^*Yaa* mice and FcγRIIB^−/−^ mice on a C57BL/6 background (provided by H. Amano at Juntendo University and S. Hirose at Toin University of Yokohama, respectively) were bred and maintained at the animal facility in the Juntendo University School of Medicine. All FcγRIIB^−/−^*Yaa* mice used in the analyses were male because only male mice carry the *Yaa* mutation. NZB mice and NZW mice were purchased from Japan SLC (Hamamatsu, Japan), and female F1 offspring were used as NZB/NZW mice. Female MRL/Fas^lpr^ (MRL/*lpr*) and MRL/Fas^+/+^ (MRL^+/+^) mice were purchased from Japan SLC. Mice were housed under specific pathogen-free conditions. Lupus-prone FcγRIIB^−/−^*Yaa* mice, NZB/NZW mice, and MRL/*lpr* mice were sacrificed at 16, 28, and 12 weeks of age, respectively. At these timepoints, they had developed nephritis, which was confirmed by proteinuria analyzed using a DCA Microalbumin/Creatinine Urine Test (Siemens, Erlangen, Germany), and histological analysis. All animal experiments were performed in accordance with the guidelines of laboratory animal experimentation at Juntendo University School of Medicine.

### Brain immune cell isolation

Mice were decapitated after deep anesthetization and transcardial perfusion with PBS. Brains were mechanically dissociated and enzymatically digested with collagenase D and DNase (both from Roche, Basel, Switzerland), dissolved in RPMI 1640 medium (Thermo Fisher Scientific, Waltham, MA, USA) supplemented with 10% fetal bovine serum, 2 mM l-glutamine, 50 U/mL penicillin, and 50 μg/mL streptomycin (all from Thermo Fisher Scientific). Digested brain tissue was suspended in 30% Percoll (GE Healthcare, Chicago, IL, USA) in PBS and overlaid on a 70% Percoll layer. After centrifugation, cells in the intermediate layer were collected and washed, then used for flow cytometry analysis and cell sorting.

### Flow cytometry

For flow cytometry analysis, isolated brain cells were pre-incubated for 15 min with purified anti-mouse CD16/32 (BioLegend, San Diego, CA, USA) to block Fc-mediated non-specific binding of antibodies. Then, cells were stained with the following antibodies: anti-CD45-BV421, anti-CD3ε-PE/Dazzle 594, anti-CD19-PE, anti-CD4-PerCP/Cy5.5, anti-CD8a-PE/Cy7, anti-CD69-APC/Cy7, anti-H-2-FITC, anti-CD317-PE, anti-Ly6C-APC-Cy7, anti-I-A/I-E-BV605 (all from BioLegend), anti-CD11b-AF700 (BD Biosciences, Franklin Lakes, NJ, USA), anti-CD45R/B220-APC (Thermo Fisher Scientific), and anti-CD11c-PE/Cy7 (Tonbo Biosciences, San Diego, CA, USA) for 20 min on ice. After surface staining, dead cells were stained with Zombie Aqua™ Fixable Viability Kit (BioLegend).

Data were acquired on a FACS LSR Fortessa (BD Biosciences), and the percentage of each cell population and mean fluorescence intensity were analyzed using FlowJo software (TreeStar Inc., Ashland, OR, USA). For the analysis of cell numbers of a specific population, total brain cells were visually counted after density gradient centrifugation as described above; then, actual numbers were calculated using the percentages from flow cytometric analysis.

### Immunohistochemistry

For the immunohistological staining of Iba-1, CD3, GFAP, and MHC class I, mice were transcardially perfused with 4% paraformaldehyde/PBS (4% PFA), and then, the brains were removed. After additional fixation with 4% PFA for 12–24 h at 4 °C, the brains were cryo-protected in 30% sucrose/PBS at 4 °C. Then, the brains were embedded in OCT Compound (Sakura Fintek Japan, Tokyo, Japan) and frozen in liquid nitrogen. Ten-micrometer cryostat sections were stained with the following antibodies: rabbit anti-iba1 (Wako, Osaka, Japan), rabbit-anti-CD3 (Abcam, Cambridge, UK), goat anti-GFAP (Santa Cruz Biotechnology, Dallas, TX, USA), and rat anti-MHC class I (Abcam) followed by biotinylated secondary antibodies: anti-rabbit IgG (Vector Laboratories, Burlingame, CA, USA), anti-goat immunoglobulins (Agilent, Santa Clara, CA, USA), or anti-rat IgG (Vector Laboratories). Horseradish peroxidase (HRP)-conjugated streptavidin (Agilent) and diaminobenzidine (DAB) were used for visualization, and hematoxylin was used as a counterstain. For the fluorescent staining of CD11b and CD11c, mouse brains were removed after transcardial perfusion with PBS and then frozen with liquid nitrogen after being embedded in OCT compound. Ten-micrometer cryostat sections were fixed in acetone and stained with rat anti-CD11b and Armenian hamster anti-CD11c (both TONBO Biosciences), followed by AF 488-conjugated anti-rat IgG (Jackson ImmunoResearch, West Grove, PA, USA), biotinylated anti-hamster IgG (Vector Laboratories), and AF 594-conjugated streptavidin (Thermo Fisher Scientific). Staining of sections was visualized with a fluorescence microscope (BZ-X700; Keyence, Osaka, Japan).

### Microglia sorting

Brains of transcardially perfused mice were enzymatically digested, and microglia, identified as CD11b^+^CD45^int^ cells, were sorted from batches of mice (from 2 to 3 mice for qRT-PCR analysis and from 5 to 8 mice for RNA-seq analysis) using a FACSAria Fusion cell sorter (BD Biosciences).

### RNA-seq analysis

Total RNA was isolated from sorted microglia using an RNeasy Micro Kit (Qiagen, Hilden, Germany) and further purified using NucleoSpin RNA XS (Takara Bio Inc., Kusatsu, Japan/Macherey-Nagel, Düren, Germany). RNA-seq libraries were generated with the Ovation SoLo RNA-Seq System, Mouse kit (NuGEN, Redwood City, CA, USA) using 5 ng of total RNA. The cDNA libraries were sequenced by 50-base single-read sequencing on an Illumina HiSeq 2500 sequencer (Illumina, San Diego, CA, USA). The sequencing run and the base call analysis were performed according to the HiSeq 2500 System Guide with TruSeq SBS kit v3-HS. After sequencing, raw sequence data were generated with processing by CASAVA-1.8.4 with version RTA 1.17.20.0. Reads were mapped to the mm10 genome with tophat2. Two base mismatches were allowed for the mapping. Normalized FPKM values and differential gene expression analyses were generated with cuffdiff2. *Q* values (Benjamini-Hochberg correction) lower than 0.05 were considered significant. Gene ontology enrichment analysis was performed using GOseq. Results were visualized using CateGOrizer [23]. Heatmaps were generated using heatmap.2 in the gplots package of R.

### Morphological analysis of microglia

For the morphological analysis of microglia, 40-μm cryostat sections were stained as free-floating sections with rabbit anti-Iba1 (Wako, Osaka, Japan) for 48 h followed by AF 488-conjugated anti-rabbit IgG (Jackson ImmunoResearch, West Grove, PA, USA) for 4 h. Confocal images of microglia were made with Leica TCS-SP5 (Leica microsystems, Wetzler, Germany). Ten-micrometer thick images constructed from 20 planes along the *z*-axis were obtained from hippocampal areas. The morphology of each microglia was analyzed using ImageJ software [24]. Among 20 planes obtained, one plane that crossed the midpoint of the cell body was analyzed for the following three parameters: cell soma size was measured as a two-dimensional area of cell bodies except for their processes, diameter of primary processes was measured as the averaged thickness of the three largest primary processes of each cell, and the total process length was measured as the sum of the lengths of visible processes.

### LPS stimulation of microglia

Sorted microglia from two to three mice were placed in a round-bottom 96-well plate at 5 × 10^4^ cells per well in RPMI medium (supplemented with the same factors as for brain immune cell isolation). Then, PBS or LPS (Sigma-Aldrich, St. Louis, MO, USA) was added at a concentration of 50 ng/ml. Cells were incubated for 2 h at 37 °C with 5% CO_2_. Total RNA was then isolated using an RNeasy micro kit (Qiagen) according to the manufacturer’s instructions.

### Quantitative real-time polymerase chain reaction (qRT-PCR)

RNA was extracted from sorted microglia using an RNeasy micro kit (Qiagen) according to the manufacturer’s instructions. cDNA was prepared from total RNA by reverse transcription with ReverTra Ace qPCR RT Master Mix (Toyobo, Osaka, Japan). qRT-PCR was performed with a 7500 Fast Real-Time PCR System (Thermo Fisher Scientific) using Fast SYBR Green Master Mix (Thermo Fisher Scientific). The following primers were used: *Apoe* forward: 5′-GGCAAAGCAACCAACCCTG-3′; *Apoe* reverse: 5′-CAGTGCCGTCAGTTCTTGTG-3′; *Axl* forward: 5′-CAAGAGCGATGTGTGGTCCT-3′; *Axl* reverse: 5′-TCTCACTGTTCTCCACCCCT-3′; *Clec7a* forward: 5′-GGGATCAGAGAAAGGAAGCCA-3′; *Clec7a* reverse: 5′-AGGAAGGCAAGGCTGAGAAAA-3′; *Itgax* forward: 5′-GCGTGGAGAACTTTGATGCT-3′; *Itgax* reverse: 5′-CTTGGTGTCTCTGTGCCCTC-3′; *Ccl5* forward: 5′-CAGTCGTGTTTGTCACTCGAA-3′; *Ccl5* reverse: 5′-AGAGCAAGCAATGACAGGGA-3′; *Cxcl10* forward: 5′-CCACGTGTTGAGATCATTGCC-3′; *Cxcl10* reverse: 5′-TCACTCCAGTTAAGGAGCCC-3′; *Tnf* forward: 5′-GATCGGTCCCCAAAGGGATG-3′; *Tnf* reverse: 5′-ACTTGGTGGTTTGCTACGAC-3′; *Il6* forward: 5′-CACTTCACAAGTCGGAGGCT-3′; *Il6* reverse: 5′-CTGCAAGTGCATCATCGTTGT-3′; *Gapdh* forward: 5′-GCAAGGACACTGAGCAAGAGA-3′; and *Gapdh* reverse: 5′-AGGCCCCTCCTGTTATTATG-3′. Results were normalized to *Gapdh*. Fold-changes in gene expression were calculated using the 2^−ΔΔCt^ method.

### Behavioral studies

The forced swim test and the tail-flick test were conducted. Mice were placed in an examining room 30 min before the examinations to habituate them to the environment.

The forced swim test was similar to that described elsewhere [25]. Mice were placed individually into glass cylinders (height 25 cm, diameter 10 cm) containing 10 cm of water maintained at room temperature and remained there for 6 min. A mouse was judged to be immobile when it floated in an upright position and made only small movements to keep its head above water. Time of immobility was recorded during the last 4 min of the 6-min testing period, after 2 min of habituation.

For the tail-flick test, mice were gently wrapped with paper towels and their movement was restricted except for their tails which were placed outside the wrap and freely mobile. Mice were placed on a table with their tails on a hotplate heated to 55 °C. The time between the placement of the tails and the onset of withdrawal responses (tail-flick) was measured as tail-flick latency. Tests were performed twice on separate days, and the mean values were calculated.

### Statistical analysis

Statistical analyses (except for RNA-seq) were performed using Prism software (Graphpad, La Jolla, CA, USA). Significance was determined by Student’s *t* test or one-way ANOVA followed by the post-tests described in the figure legends.

## Results

### Analysis of lymphocyte populations in the CNS of lupus-prone mice

**Fig. 1:**
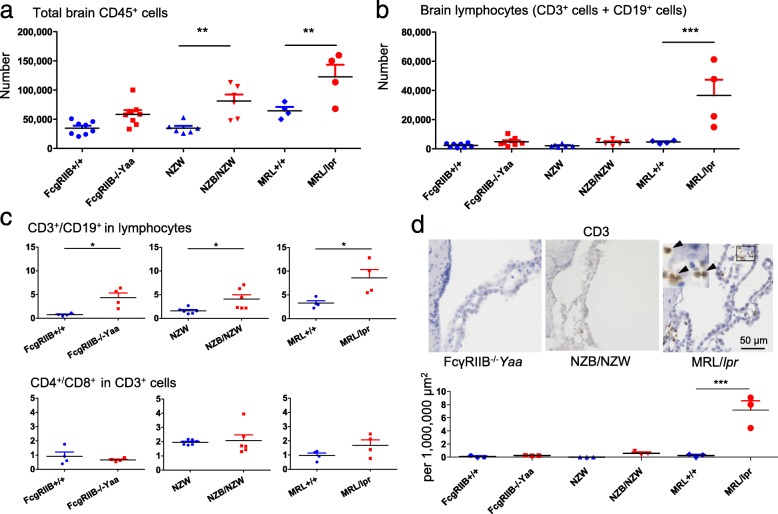
Altered immune cell composition in the brains of lupus model mice. **a** Numbers of total brain CD45^+^ cells in three lupus models and controls analyzed by flow cytometry. **b** Number of total brain lymphocytes, defined as CD3^+^ cells and CD19^+^ cells in CD45^+^ cells, in three lupus models and controls. **c** CD3^+^/CD19^+^ ratio in lymphocytes and CD4^+^/CD8^+^ ratio in CD3^+^ cells. **d** Immunostaining of CD3^+^ cells in the choroid plexus. Magnification of the boxed area is shown in the upper left of MRL/*lpr*. Arrowheads indicate CD3^+^ cells. Images are representative of *n* = 3 comparisons in each lupus model and controls. Numbers of CD3^+^ cells were counted and shown as number per mean area of the choroid plexus. In **a**, **b**, and **c**, number or ratio of cells was analyzed by flow cytometry. Symbols represent individual mice (*n* = 8 for **a** and **b**, *n* = 4 for C for FcγRIIB^−/−^*Yaa* mice and controls, *n* = 6 for NZB/NZW mice and controls, *n* = 4 for MRL/*lpr* mice and controls) and horizontal lines indicate the mean and SEM. In **a**, **b**, and **d**, **P < 0.01, and ***P < 0.01 by Bonferroni’s multiple comparison test. In **c**, *P < 0.05, **P < 0.01, and ***P < 0.01 by Student’s *t* test

MRL/*lpr* and NZB/NZW mice are well-established spontaneous models of SLE [18]. FcγRIIB^−/−^*Yaa* mice are a genetically modified mouse strain that lack the suppressing Fcγ receptor FcγRIIB, and have a duplication of TLR7 provided by the *Yaa* gene [21]. These mice spontaneously exhibit clinical and serological features comparable with human SLE. Among them, MRL/*lpr* mice are the most extensively investigated, and their CNS pathology and marked infiltration of lymphocytes into the choroid plexus were previously demonstrated [18]. These three mouse strains spontaneously developed nephritis, which was confirmed by proteinuria and the histological analysis of kidneys (Additional file 1: Figure S1 A and B). We investigated whether immune cells were altered in the brains of these lupus-prone mice. We harvested mononuclear cells from brains by enzymatic digestion and density gradient separation and analyzed CD45^+^ immune cells by flow cytometry (Additional file 2: Figure S2). CD45^+^ immune cells were increased in MRL/*lpr* mice and NZB/NZW mice (Fig. 1a) compared with the other groups, and there was a tendency for them to be increased in FcγRIIB^−/−^*Yaa* mice. We compared the number of CD3^+^ and CD19^+^ lymphocytes among CD45^+^ cells (Fig. 1b, Additional file 3: Figure S3). Consistent with a previous study [20], the number of lymphocytes infiltrating into the brain was markedly increased in MRL/*lpr* mice compared with control mice. In contrast, the increase of lymphocytes in the brains of NZB/NZW mice and FcγRIIB^−/−^*Yaa* mice was not obvious. The ratio of CD3^+^/CD19^+^ cells analyzed by flow cytometry was increased in all three lupus models indicating lymphocytes in the brains were T cell dominant with a similar CD4^+^/CD8^+^ ratio to controls (Fig. 1c). Using immunohistochemistry, we confirmed that CD3^+^ cells were observed in the choroid plexus of MRL/*lpr* mice but not the other lupus-prone mice (Fig. 1d). We then assessed the activation status of lymphocytes by their expression of CD69. In FcγRIIB^−/−^*Yaa* mice, numbers of CD69^+^ cells were increased in the CD3^+^ and CD19^+^ cell populations (Additional file 4: Figure S4). In NZB/NZW mice, numbers of CD69^+^ cells tended to be increased although the difference did not reach statistical significance. In contrast, CD69^+^ cells were increased only in CD19^+^ cells but not in CD3^+^ cells in MRL/*lpr* mice, indicating that B cells were selectively activated (Additional file 4: Figure S4). These results suggested that the activation of lymphocytes was enhanced in lupus-prone mice, whereas the massive infiltration of lymphocytes into the CNS was limited to MRL/*lpr* mice.

### Analysis of innate immune cells in the CNS of lupus-prone mice

**Fig. 2:**
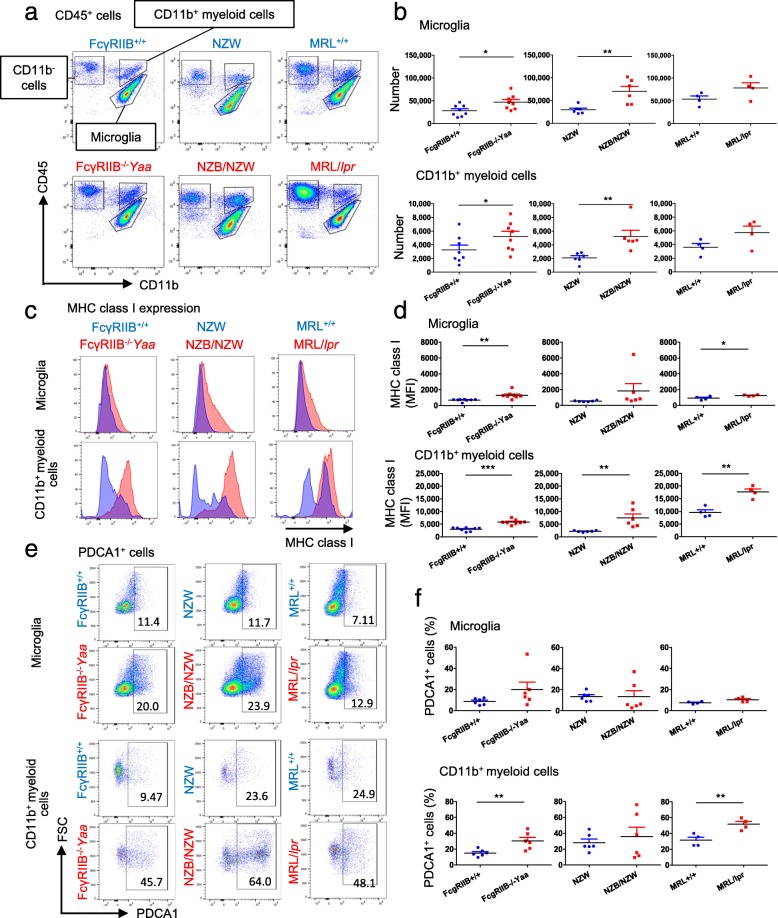
Altered number and activation status of microglia and CD11b^+^ myeloid cells in the CNS of lupus model mice. **a** Representative plots of CD45^+^ cells in brains by flow cytometry. Cells were characterized as CD11b^−^ cells, CD11b^+^ myeloid cells, and microglia. **b** Number of total brain microglia and CD11b^+^ myeloid cells. **c** Representative histograms of MHC class I expression in microglia and CD11b^+^ myeloid cells. Red histograms represent lupus model mice, and blue histograms represent their controls. **d** Comparison of MHC class I expression in lupus model mice and their controls. **e** Representative plots of PDCA1 expression in microglia and CD11b^+^ myeloid cells. Percentages of positive cells are shown. **f** Comparison of PDCA1-positive cells in lupus model mice and their controls. In **b**, **d**, and **f**, symbols represent individual mice (*n* = 8 for B and D, *n* = 6 for F for FcγRIIB^−/−^*Yaa* mice and controls, *n* = 6 for NZB/NZW mice and controls, *n* = 4 for MRL/*lpr* mice and controls) and horizontal lines indicate the mean and SEM. *P < 0.05, **P < 0.01, and ***P < 0.01 by Student’s *t* test

Flow cytometry analysis of brain immune cells indicated that brain CD45^+^ cells could be characterized into three populations by their expressions of CD11b and CD45 (Fig. 2a, the gating strategy is shown in Additional file 2: Figure S2). CD11b^+^ cells comprised mononuclear phagocytes and were divided further into two populations. CD11b^+^CD45^int^ cells are microglia that reside in the brain parenchyma [9], and CD11b^+^CD45^hi^ cells are mainly myeloid-derived macrophages that reside at the interfaces of the brain and periphery [9]. Hereafter, the CD11b^+^ CD45^hi^ population is referred to as CD11b^+^ myeloid cells. Numbers of microglia and CD11b^+^ myeloid cells were increased in all three lupus-prone mouse strains (Fig. 2b). We next examined the activation status of microglia and CD11b^+^ myeloid cells. Because there was no increased expression of CD69 on microglia or CD11b^+^ myeloid cells, we examined the expressions of MHC class I and PDCA1, which are known to increase on these cells in response to IFN stimulation [26]. MHC class I expression was increased on microglia and CD11b^+^ myeloid cells from lupus-prone mice but not on microglia from NZB/NZW mice, which was not significant (Fig. 2c, d). The expression of PDCA1 was also increased on CD11b^+^ myeloid cells from FcγRIIB^−/−^*Yaa* and MRL/*lpr* mice. Although the difference in PDCA1 expression on microglia was not statistically significant between lupus-prone mice and control mice, there was a tendency for it to be increased in FcγRIIB^−/−^*Yaa* mice (Fig. 2e, f). These results suggested the activation of innate immune cells in lupus brains.

### Expansion of Ly6C^lo^ cells in CD11b^+^ myeloid cells in the CNS

**Fig. 3:**
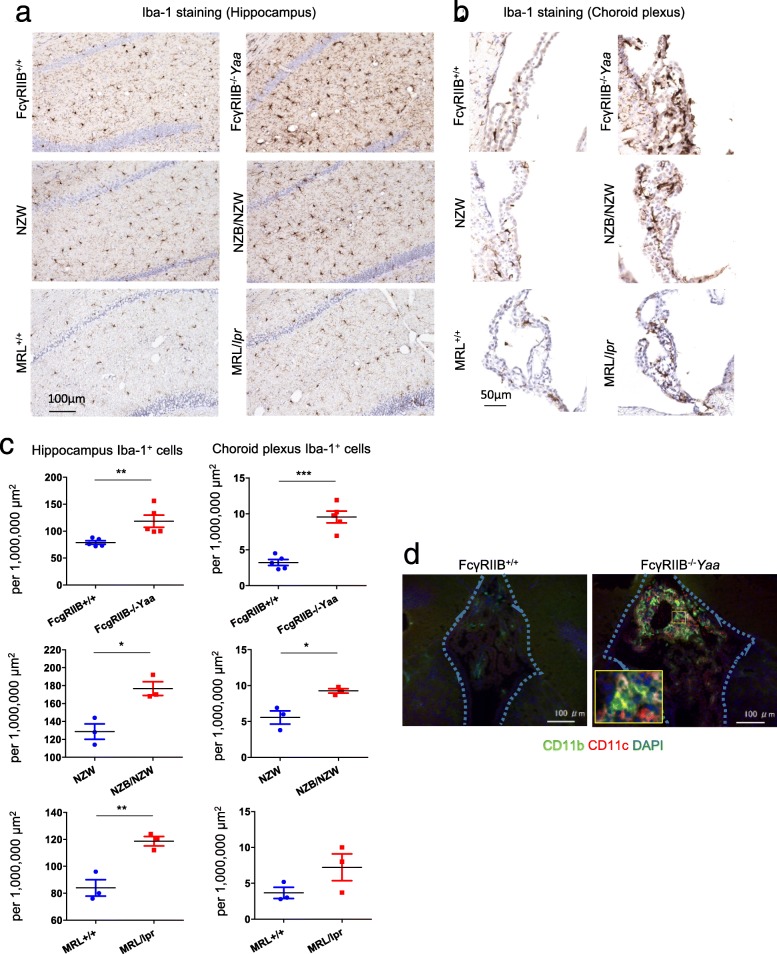
Immunohistology of brain microglia and myeloid cells. **a** Representative images of Iba-1 staining in the hippocampus of lupus model mice and their controls. **b** Representative images of Iba-1 staining in the choroid plexus (lateral ventricles) of lupus model mice and their controls. **c** Comparison of the numbers of Iba-1-positive cells in the hippocampus and choroid plexus of lupus model mice and their controls. **d** CD11b^+^ cells accumulated in the choroid plexus of FcγRIIB^−/−^*Yaa* mice. Among them, CD11b^+^CD11c^+^ were increased. Areas surrounded by the blue dashed lines are the dorsal third ventricles and the choroid plexus. In **a**, **b**, and **c**, *n* = 5 for FcγRIIB^−/−^*Yaa* mice and controls, *n* = 3 for NZB/NZW mice and controls, *n* = 3 for MRL/*lpr* mice and controls. In **d**, *n* = 3 for FcγRIIB^−/−^*Yaa* mice and controls. In **c**, numbers of Iba-1^+^ cells per designated area of the hippocampus or choroid plexus were counted. Symbols represent individual mice, and horizontal lines indicate the mean and SEM. **P < 0.01, and ***P < 0.01 by Student’s *t* test

Because microglia and CD11b^+^ myeloid cells showed an activated status, we analyzed these cells further. Immunohistochemistry of lupus model mice and their controls confirmed an increase in Iba-1^+^ cell numbers in the parenchyma (Fig. 3a, c) and choroid plexus (Fig. 3b, c) of lupus model mice. Iba-1^+^ cells in the parenchyma and choroid plexus represented microglia and myeloid cells, respectively. These results were in accordance with the flow cytometry results shown in Fig. 2. Fluorescent immunostaining revealed CD11b^+^ CD11c^+^ cells were dominant among CD11b^+^ myeloid cells in the choroid plexus of FcγRIIB^−/−^*Yaa* mice (Fig. 3d).

**Fig. 4:**
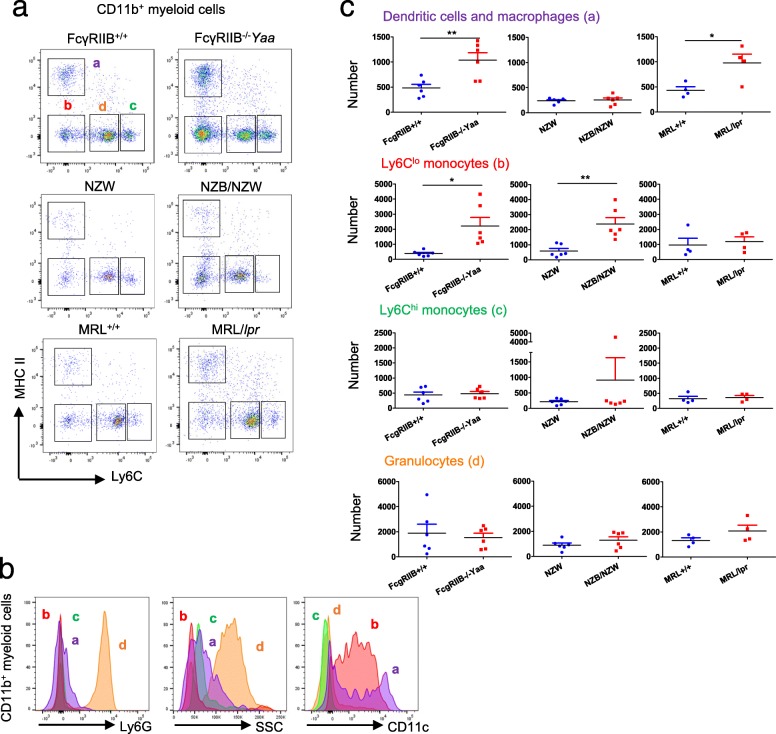
Further classification and analysis of CD11b^+^ myeloid cells. **a** Representative plots of brain CD11b^+^ myeloid cells in lupus model mice and their controls. They were classified into four populations. a (dendritic cells and macrophages): MHC II^hi^Ly6C^lo^ cells, b (Ly6C^lo^ monocytes): MHC II^lo^Ly6C^lo^ cells, c (Ly6C^hi^ monocytes): MHC II^lo^Ly6C^hi^ cells, and d (granulocytes): MHC II^lo^Ly6C^int^ cells. **b** Representative plots of Ly6G expression, side scatter (SSC) and CD11c expression in CD11b^+^ myeloid cells from FcγRIIB^−/−^*Yaa* mice**.** High Ly6G and high SSC in population “d” indicate their granulocyte character. **c** Numbers of dendritic cells and macrophages (a), Ly6C^lo^ monocytes (b), Ly6C^hi^ monocytes (c), and granulocytes (d) in three lupus models. In **c**, symbols represent individual mice (*n* = 6 for FcγRIIB^−/−^*Yaa* mice and controls, *n* = 6 for NZB/NZW mice and controls, *n* = 4 for MRL/*lpr* mice and controls) and horizontal lines indicate the mean and SEM. *P < 0.05, **P < 0.01, and ***P < 0.01 by Student’s *t* test

CD11b^+^ myeloid cells were further classified into four populations by Ly6C and MHC class II expressions by flow cytometry (Fig. 4A). MHC II^hi^ cells (a) are considered dendritic cells and macrophages [9] and MHC II^lo^ cells are assumed to be of peripheral blood origin. Ly6C^int^ cells (d) show high side scatter and express Ly6G, indicating these cells are granulocytes (Fig. 4B). Ly6C^hi^ cells (c) and Ly6C^lo^ cells (b) are considered classical and non-classical monocytes, respectively [27]. Ly6C^lo^ monocytes (b) and a population composed of dendritic cells and macrophages (a) were CD11c-positive cells (Fig. 4B). Increased number of cells in this population in FcγRIIB^−/−^*Yaa* mice coincided with an increased number of CD11b^+^CD11c^+^ cells in the choroid plexus (Figs. 3d and 4C). Ly6C^lo^ monocytes (b) were increased in FcγRIIB^−/−^*Yaa* mice and NZB/NZW mice. In contrast, Ly6C^hi^ monocytes (c) were not increased in any lupus mouse strains (Fig. 4C). Taken together, a numerical increase in microglia and myeloid cells in the CNS is a shared feature of lupus-prone mice, and a dominant increase of Ly6C^lo^ cells is penetrant among some models of lupus-prone mice.

### Gene expression analysis reveals the unique inflammatory status of microglia in lupus-prone mice

**Fig. 5:**
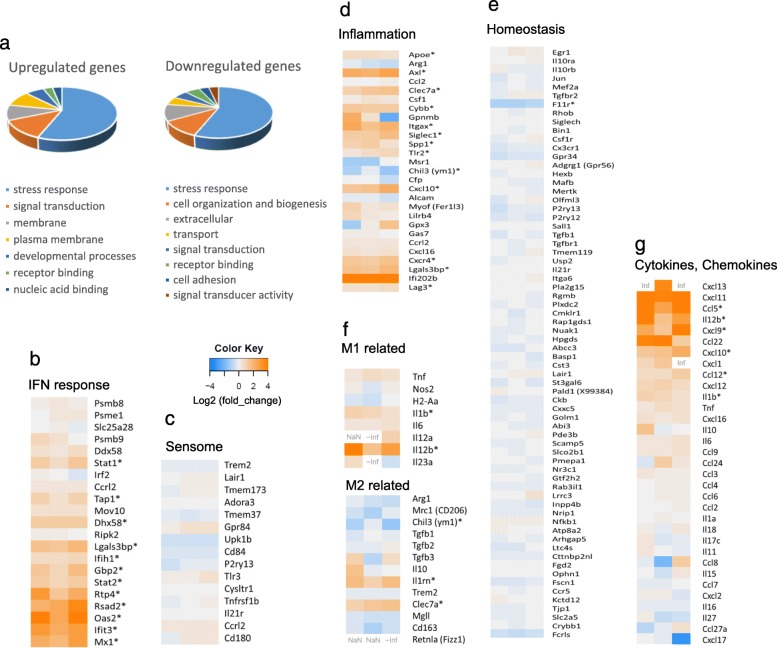
RNA-seq analysis of microglia from FcγRIIB^−/−^*Yaa* mice. **a** Visualized images of GO enrichment analysis. Top 100 GO terms enriched in upregulated or downregulated genes in microglia from FcγRIIB^−/−^*Yaa* mice were analyzed with CateGOrizer. **b**–**e** Heat map of mRNA expressions in microglia from FcγRIIB^−/−^*Yaa* mice relative to FcγRIIB^+/+^ mice were measured by RNA-seq. Relative FPKM values of FcγRIIB^−/−^*Yaa* mice compared with FcγRIIB^+/+^ mice are expressed as colored columns. Results are of pooled samples from three groups of 5 to 8 mice each analyzed individually. **b** IFN response genes. **c** Sensome genes. **d** Inflammation-related genes. **e** Homeostasis-related genes. **f** Genes related to M1 polarization and M2 polarization. **g** Cytokines and chemokines. In **b**–**g**, genes with log2 fold changes greater than four are shown with maximum color. NaN = transcripts of FcγRIIB^−/−^*Yaa* and FcγRIIB^+/+^ were not detected. -inf = transcripts of FcγRIIB^−/−^*Yaa* were not detected. Inf = transcripts of FcγRIIB^+/+^ were not detected. * = differentially expressed genes

We next examined the activation status of microglia in lupus-prone mice. We analyzed changes in gene expression in microglia by RNA-seq analysis in FcγRIIB^−/−^*Yaa* mice because this model is characterized by the augmentation of innate immune signals and immunological changes were not affected by a massive increase in lymphocytes. Microglia were isolated, and differentially expressed genes (DEG) between FcγRIIB^−/−^*Yaa* and FcγRIIB^+/+^ strains were assessed. The number of reads per sample ranged from 524 to 578 million, and 84.7–86.5% were successfully mapped onto the mm10 genome. There were 175 DEG with 144 upregulated genes and 31 downregulated genes. Gene ontology enrichment analysis revealed many DEG were related to stress responses (Fig. 5a). In a previous report, gene expression analysis of microglia from lupus-prone 564Igi mice, a B cell receptor insertion model with known autoantibody specificity, showed the upregulation of IFN response genes and 15 sensome genes, a set of genes microglia use to sense their environment [19]. In FcγRIIB^−/−^*Yaa* mice, IFN response genes, but none of the 15 sensome genes, were generally upregulated (Fig. 5b, c). Upregulated expression of MHC class I and a tendency for increased PDCA1 by flow cytometry correlated with the upregulated gene expressions of MHC class I associated genes and *Pdca1* (Additional file 5: Table S1). We also investigated neurodegeneration-related genes based on 28 inflammation-related and 68 homeostasis-related genes previously reported [11]. In microglia from FcγRIIB^−/−^*Yaa* mice, 28 inflammation-related genes were generally upregulated (Fig. 5d) including *Apoe*, *Axl*, *Clec7a*, and *Itgax*, genes relevant to “primed” microglia that do not secrete high amounts of cytokines but become hyper-reactive when triggered by proinflammatory stimuli [28]. Indeed, the expression of proinflammatory cytokine genes such as *Tnf* and *Il6* were not upregulated. Homeostasis-related genes were slightly downregulated although a statistically significant change was only observed for *F11r* (Fig. 5e). This activation status did not match classical M1 or M2 status [29, 30] (Fig. 5f). In FcγRIIB^−/−^*Yaa* microglia, the expressions of several chemokine and cytokine genes, such as *Ccl5*, a chemoattractant for monocytes and T lymphocytes [31], and *Cxcl10*, a chemoattractant for T lymphocytes, were upregulated. In contrast, there was no upregulation of *Ccl2*, a chemoattractant for Ly6C^hi^ classical monocytes (Fig. 5g). These results were coincident with the T cell dominated infiltration of lymphocytes and Ly6C^lo^ cell dominated infiltration of monocytes. Of note, CCR5, the receptor for CCL5, was expressed at a lower level in Ly6C^hi^ monocytes compared with Ly6C^lo^ monocytes (Additional file 6: Figure S5).

**Fig. 6:**
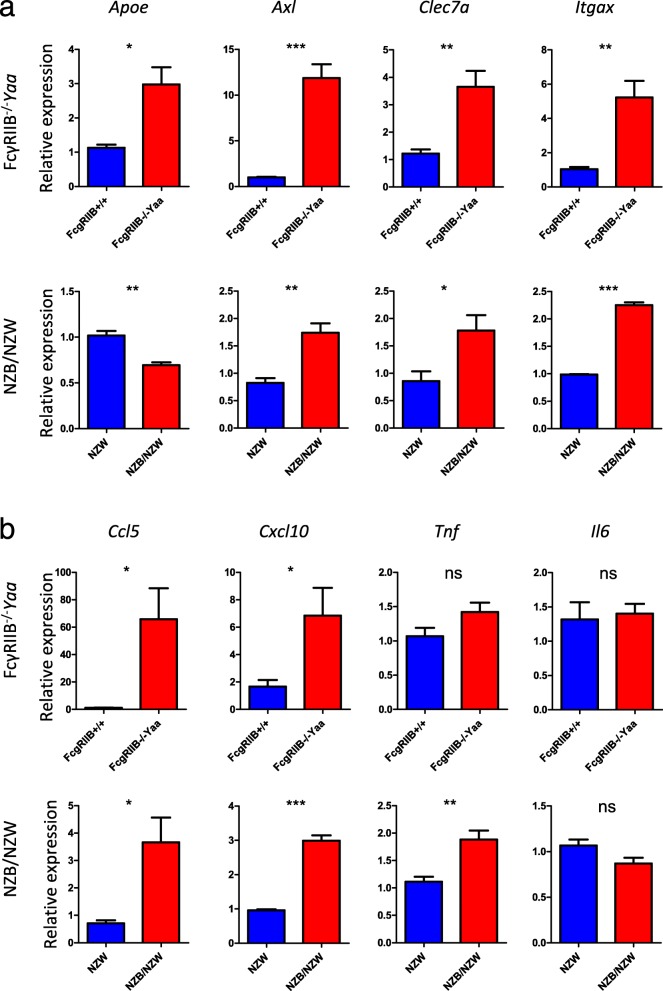
qRT-PCR analysis of inflammation-related genes in microglia from FcγRIIB^−/−^*Yaa* mice and NZB/NZW mice. **a** Expression of neurodegeneration related genes. **b** Expression of chemokines and proinflammatory cytokines. In **a** and **b**, RNA extracted from sorted microglia from two to three mice per experiment was used for qRT-PCR analysis. Results are of pooled samples from four groups of 2 to 3 mice each analyzed individually. Horizontal lines indicate the mean and SEM. *P < 0.05, **P < 0.01, and ***P < 0.01, ns = no statistical significance, by Student’s *t* test

We then assessed whether gene expression changes in microglia from FcγRIIB^−/−^*Yaa* mice were shared in another lupus-prone mouse strain, NZB/NZW. We analyzed the expressions of genes characteristically upregulated in the RNA-seq analysis of FcγRIIB^−/−^*Yaa* mice. Upregulated expressions of *Axl*, *Clec7a*, and *Itgax* were observed in FcγRIIB^−/−^*Yaa* mice and NZB/NZW mice although *Apoe* was not upregulated in NZB/NZW mice (Fig. 6a). The upregulation of *Ccl5* and *Cxcl10* expressions was also observed in FcγRIIB^−/−^*Yaa* mice and NZB/NZW mice. In terms of inflammatory cytokines, *Tnf* was upregulated in NZB/NZW mice but the relative expression was within a two-fold change compared with NZW mice. *Il6* was not upregulated in either mouse strain (Fig. 6b). Overall, microglia from lupus-prone mice exhibited a unique polarizing status characterized by the upregulation of neurodegeneration-related genes in addition to IFN response genes. Some of the characteristic genes upregulated in FcγRIIB^−/−^*Yaa* mice were similarly upregulated in microglia from NZB/NZW mice.

### Morphological changes and hypersensitivity to LPS indicates the primed status of microglia in FcγRIIB^−/−^*Yaa* mice

**Fig. 7:**
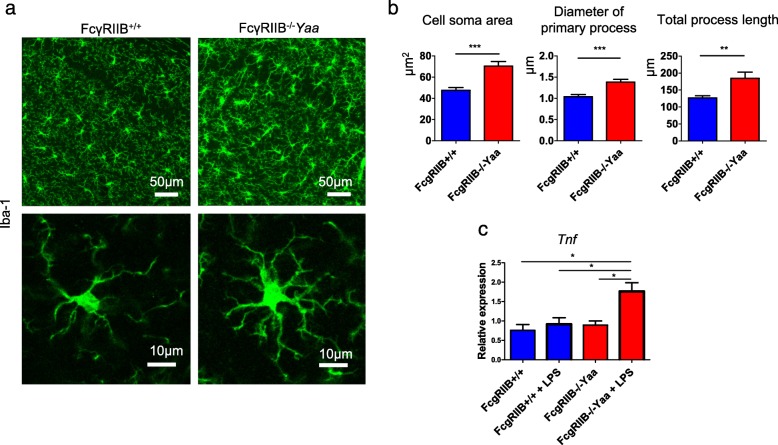
Analysis of the primed status of microglia from FcγRIIB^−/−^*Yaa* mice. **a**, **b** Confocal microscope images of microglia from FcγRIIB^−/−^*Yaa* mice revealed they had larger cell soma and hypertrophic processes (*n* = 3 for FcγRIIB^−/−^*Yaa* mice and controls). **c** Relative expressions of *Tnf* and *Il6* genes after stimulation with a small amount of LPS. In **b**, data from three representative cells in three FcγRIIB^−/−^*Yaa* mice and three FcγRIIB^+/+^ mice were obtained, compared, and analyzed by Student’s *t* test. In **c**, sorted microglia from two to three mice per experiment were used for the experiments. Sum of three experiments was compared and analyzed by one-way ANOVA and post hoc Tukey’s multiple comparison test. *P < 0.05, **P < 0.01, and ***P < 0.01

The gene expression pattern of microglia from FcγRIIB^−/−^*Yaa* mice indicated their primed status rather than their acute inflammatory status. To confirm the primed status of microglia, we analyzed microglia from FcγRIIB^−/−^*Yaa* mice by their morphology and response to proinflammatory stimuli. Confocal microscope analysis revealed microglia from FcγRIIB^−/−^*Yaa* mice had a larger cell soma and hypertrophic processes compared with control mice (Fig. 7a). This morphology was analogous to primed microglia observed in accelerated aging [22]. Microglia from FcγRIIB^−/−^*Yaa* mice showed the upregulated expression of *Tnf* after stimulation with a small amount of LPS, which did not affect control microglia, further suggesting the primed status of microglia from FcγRIIB^−/−^*Yaa* mice (Fig. 7b).

### Immune activation in the brains of FcγRIIB^−/−^*Yaa* mice is accompanied by reactive responses of brain intrinsic cells and behavioral changes

**Fig. 8:**
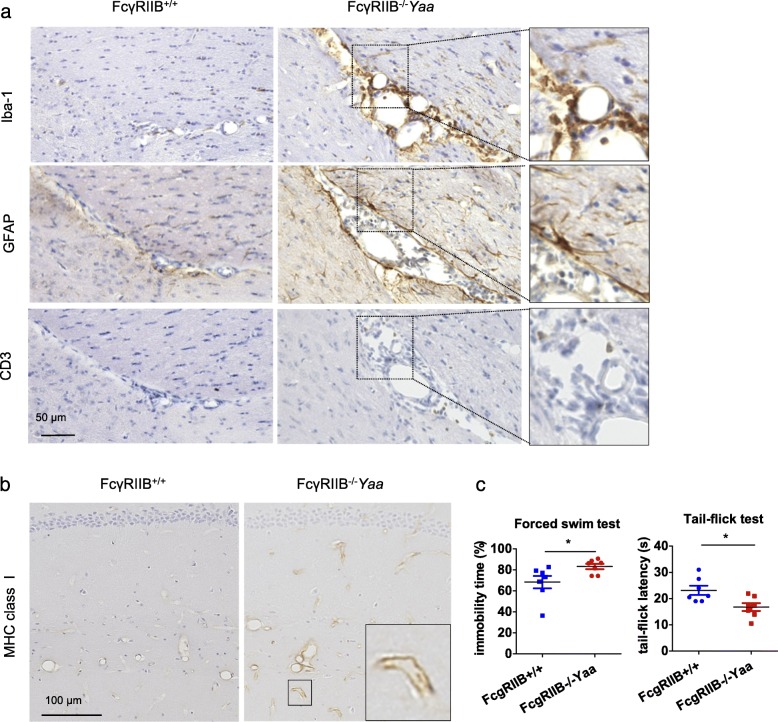
Innate immune responses in the brains of FcγRIIB^−/−^*Yaa* mice and effects of immunological changes on behavior. **a** Around the perivascular area of the meningeal space (hippocampal sulcus) where Iba-1^+^ myeloid cells accumulated, GFAP-positive reactive astrocytes and CD3^+^ cells were also increased. **b** MHC class I is upregulated in the vascular endothelium of FcγRIIB^−/−^*Yaa* mice (hippocampus). **c** Results of the forced swim test and the tail-flick test in FcγRIIB^−/−^*Yaa* mice and their control mice. **a** and **b** are representative images of *n* = 5 experiments of lupus models and controls. In **c**, symbols represent individual mice (*n* = 7) and horizontal lines indicate the mean and SEM. *P < 0.05 by Student’s *t* test

In FcγRIIB^−/−^*Yaa* mice, immunohistochemical analysis at the interfaces of the CNS and periphery, such as the perivascular area of meninges, revealed Iba-1^+^ myeloid cells were accompanied by reactive astrocytes with highly positive GFAP staining [13], which was accompanied by a slight accumulation of CD3^+^ T cells (Fig. 8a). In addition, vascular endothelium upregulated the expression of MHC class I (Fig. 8b), suggesting the reactive response of these cells upon innate cell activation.

Finally, we assessed whether immunological changes observed in the brain of FcγRIIB^−/−^*Yaa* mice were related to behavioral changes. We conducted the forced swim test and the tail-flick test, which examine depressive-like behavior and heat hyperalgesia, respectively, both of which were shown to be increased in lupus models [32] [33]. FcγRIIB^−/−^*Yaa* mice had an increased immobility time and shortened tail-flick latency compared with controls (Fig. 8c), indicating they were more depressive and hyperalgesic. Taken together, immunological changes in the brain of FcγRIIB^−/−^*Yaa* mice involved brain intrinsic cells, such as uniquely activated microglia, astrocytes, and vascular endothelium as well as peripheral immune cells. These immunological changes were accompanied by neuropsychiatric symptoms.

## Discussion

In the present study, we investigated immune cells in the CNS of three murine models of SLE and revealed that an increase in the number and activation status of innate immune cells in the CNS was a shared feature of lupus-prone mice. Gene expression analysis demonstrated that microglia from FcγRIIB^−/−^*Yaa* mice exhibited a unique activation status characterized by the upregulation of neurodegeneration-related genes and IFN response genes.

A previous study [20] reported the massive infiltration of lymphocytes in the CNS of MRL/*lpr* mice, which was characterized by lymphoproliferation caused by a *Fas* mutation [20]. In accord, we observed an increase in lymphocytes in the CNS of MRL/*lpr* mice. However, we did not observe an increase in the number of infiltrating lymphocytes in the CNS of NZB/NZW and FcγRIIB^−/−^*Yaa* mice. The lupus-like disease in MRL/*lpr* mice was reported to be aggravated by a deficiency in *Ifnar1*, a gene encoding IFN receptors for type I IFNs [34]. This suggests that MRL/*lpr* mice have a unique pathogenesis different from other lupus models or human SLE, where type I IFNs are critical for pathogenesis [1, 35]. Indeed, a mutation in *FAS* causes autoimmune lymphoproliferative syndrome, a different clinical disease from SLE [36]. In contrast, the activation of innate immune cells including myeloid cells and microglia is a shared feature among all three lupus-prone mouse strains. Even in MRL/*lpr* mice, neuropsychiatric manifestations and monocyte infiltration in the choroid plexus remained after bone marrow transplantation [32], indicating the importance of innate immune cells in the pathogenesis of NPSLE, independent of adaptive immunity.

In inflammatory conditions of the CNS, blood monocytes are recruited from the circulation and differentiate into monocyte-derived macrophages [37]. In acute inflammatory conditions such as infection or trauma, Ly6C^hi^ monocytes, which are the mouse counterparts of human CD14^+^ classical monocytes, are recruited to the site of inflammation via CCL2-CCR2 interactions [27]. Under sterile inflammatory conditions in the CNS such as status epileptics and experimental autoimmune encephalomyelitis (EAE), Ly6C^hi^ monocytes are recruited to the CNS via CCL2-CCR2 interactions [38, 39]. Although microglia were reported to be important for the production of CCL2 during acute inflammation [38], the level of *Ccl2* expression was not increased in FcγRIIB^−/−^*Yaa* mice in the present study, suggesting the polarizing status of microglia during lupus is different from that in acute inflammation. Consistent with that finding, Ly6C^lo^ cells, which are the mouse counterparts of human CD16^+^ non-classical monocytes, dominantly accumulated in the CNS of FcγRIIB^−/−^*Yaa* and NZB/NZW mice. Ly6C^lo^ monocytes in the steady-state maintained the integrity of vascular endothelium and protected mice from viral infection [40]. These cells were also recruited to chronic inflammatory sites and are likely to be involved in disease pathogenesis [41–43]. In a murine model of arthritis, Ly6C^lo^ monocytes accumulated in inflamed joints, and osteoclasts were differentiated from these monocytes [41]. Furthermore, increased numbers of Ly6C^lo^ monocytes accumulated in the kidneys of murine lupus models [42, 43]. Moreover, the increase of non-classical CD16^+^ monocytes in the peripheral blood and their infiltration into the nephritis glomerulus were reported in patients with SLE [44, 45]. Ly6C^lo^ monocytes and CD16^+^ monocytes lack CCR2 but express CCR5 and CX3CR1 chemokine receptors [46, 47]. In atherosclerosis and murine lupus nephritis models, Ly6C^lo^ monocytes were recruited to lesions independent of CX3CR1 [43, 47]. These findings suggest that CX3CR1 is dispensable and that CCR5 is more important for the infiltration of Ly6C^lo^ monocytes to target tissues. Gene expression analysis revealed the upregulation of *Ccl5* transcripts in the microglia of FcγRIIB^−/−^*Yaa* mice and NZB/NZW mice, suggesting CCL5-CCR5 interactions may be important for the infiltration of Ly6C^lo^ monocytes into the brain. Indeed, increased levels of CCL5 in the serum and cerebrospinal fluid of SLE patients have been reported [48–50], and increased levels of CCL5 in the cerebrospinal fluid were not decreased even after treatment although other chemokines were decreased [51]. From these findings, CCL5 appears to have an important role in chronic insidious inflammation.

Changes in the gene expression of microglia from FcγRIIB^−/−^*Yaa* mice indicated that these cells have a unique inflammatory status. The strong upregulation of IFN-responsive genes in microglia from FcγRIIB^−/−^*Yaa* mice was consistent with the microglial gene expression analysis of 564Igi mice, a B-cell receptor insertion model [19]. Type I IFNs have different effects on the inflammatory status of the CNS. They ameliorate inflammation via effects on astrocytes and are used for the treatment of multiple sclerosis [52]. In contrast, type I IFNs were reported to be detrimental in aging brains and Alzheimer’s brains and caused microglia to engulf neuronal and synaptic debris leading to neuronal loss in 564Igi mice [19, 53, 54]. Although an IFN signature is common among lupus-prone mice, the 15 sensome genes used by microglia to sense their environment were upregulated in 564Igi mice but not in FcγRIIB^−/−^*Yaa* mice. This difference may be explained by differences in the CNS inflammatory status between the SLE models. In the analysis of 564Igi mice, microglia were harvested at relatively early timepoints before systemic autoimmunity developed, and no infiltration of immune cells was observed in the brain. Thus, effects on microglia were mostly via IFN-α in 564Igi mice. In contrast, our study obtained microglia from FcγRIIB^−/−^*Yaa* mice that had already developed lupus disease, and thus, their phenotype may reflect the lupus brain.

Microglia from FcγRIIB^−/−^*Yaa* mice also showed a gene expression pattern similar to that in neurodegenerative diseases. Reports of a microglial phenotype related to neurodegeneration include disease-associated microglia (DAM) [10], microglia with a neurodegenerative phenotype (MGnD) [11], and Dark microglia [12]. Neurodegenerative phenotypes of microglia have been observed in humans and murine models of neurodegenerative or neuroinflammatory diseases, such as Alzheimer’s disease, amyotrophic lateral sclerosis, multiple sclerosis, and aging. A common finding in these microglia is the upregulation of inflammation-related genes and the downregulation of homeostasis-related genes in addition to upregulated phagocytic activity. These features were also observed in primed microglia that were ready to become hyper-reactive when triggered by proinflammatory stimuli [28] [55], but which are different from M1 or M2 polarized inflammatory microglia. The morphological changes and hyperreactivity to LPS in microglia from FcγRIIB^−/−^*Yaa* mice demonstrated they had a primed status and were phenotypically different from microglia in acute inflammatory conditions [22, 56]. The immunopathology observed in FcγRIIB^−/−^*Yaa* mice and NZB/NZW mice had similarities including microglial gene expression. Inflammation-related genes *Axl*, *Clec7a*, *Itgax*, *Ccl5*, and *Cxcl10* were similarly upregulated although the expression of *Apoe* was different. Previous studies reported the upregulation of *Apoe* in microglia isolated from the neurodegenerative conditions described above. One explanation for this is the condition of control NZB/NZW mice. NZW mice are used as controls, but they are not normal mice. Therefore, *Apoe* overexpression might be characteristic of NZW mice. Another explanation might be the upregulation of *Apo*e is not essential for lupus CNS pathology.

Taken together, microglia in lupus-prone mice have a unique low-grade chronic inflammatory status, which we termed LAM. Innate immune activation in lupus-prone mice appeared to involve resident CNS cells such as astrocytes and vascular endothelium [13, 14], and these cells are likely to further orchestrate the chronic inflammatory status of NPSLE. Furthermore, it is important to note that neuroinflammation is affected by systemic inflammation. Neurodegenerative diseases are aggravated under the influence of systemic inflammation [16, 17]. In our study, peripheral immune cells in the CNS such as Ly6C^lo^ monocytes were increased in lupus-prone mice and they were likely to be involved in the neuropathology.

## Conclusions

We demonstrated that the activation of innate immune cells is a common feature in lupus-prone mice. These cells were characterized by a chronically activated status in addition to the upregulation of IFN-regulated genes. This unique inflammatory change in the brain may account for the chronic non-fatal but disabling neurological status such as cognitive impairment and mood disorders in human SLE. A recently reported improvement of fatigue and cognitive impairment by treatment for SLE supports the idea that neuropsychiatric symptoms recognized as not attributable to SLE have immune-related mechanisms and that the correct treatment might ameliorate them [57, 58]. Further insights into the innate immune-related mechanisms of NPSLE will lead to the development of novel therapeutic strategies.

## Supplementary information

**Additional file 1: Figure S1.** Nephritis was observed in three lupus models at the time of brain immunopathological analysis. **(A)** Proteinuria was observed in lupus models. **(B)** Representative images of PAS staining of the glomerulus in lupus model mice and their controls.

**Additional file 2: Figure S2.** Gating strategy to analyze CD45^+^ cells from whole brain cells. Representative gating strategy for FcγRIIB^-/-^*Yaa* mice is shown**. (A)** Mononuclear cells were selected by size and granularity. **(B, C)** Doublet cells were excluded. **(D)** Live CD45^+^ cells were selected. **(E)** Populations shown in this plot were used for further analysis.

**Additional file 3: Figure S3.** Gating strategy to analyze lymphocytes. Representative gating strategy for the analysis of three lupus models and controls is shown.

**Additional file 4: Figure S4.** CD69 expression in lymphocytes from lupus prone mice. CD69 expression was analyzed by flow cytometry in three lupus prone mouse strains. **(A)** Representative plots of CD69 expression in CD3^+^ lymphocytes. **(B)** Representative plots of CD69 expression in CD19^+^ lymphocytes. **(C, D)** Comparison of CD69^+^ cells between lupus model mice and their controls. In **C** and **D**, symbols represent individual mice (*n*=6 for FcγRIIB^-/-^*Yaa* mice and controls, n=6 for NZB/NZW mice and controls, *n*=4 for MRL/*lpr* mice and controls) and horizontal lines indicate the mean and SEM. *P < 0.05, **P < 0.01, and ***P < 0.01 by Student’s *t*-test.

**Additional file 5: Table S1.** Expressions of PDCA1 and MHC class I genes on RNA-seq analysis of microglia in FcγRIIB^-/-^*Yaa* mice and their controls.

**Additional file 6: Figure S5.** Expression of CCR5 in brain monocytes. Expression of CCR5 was examined by flow cytometry in FcγRIIB^-/-^*Yaa* mice. **(A)** Representative histograms of CCR5 expression in Ly6C^hi^ monocytes and Ly6C^lo^ monocytes. Staining of isotype controls is shown. **(B)** Comparison of CCR5 positive cells in Ly6C^hi^ monocytes and Ly6C^lo^ monocytes. Higher numbers of CCR5 positive cells were present in Ly6C^lo^ monocytes compared with Ly6C^hi^ monocytes. In **B**, Symbols represent individual mice (*n*=3) and horizontal lines indicate the mean and SEM. *P < 0.05, by Student’s *t*-test.

## Data Availability

The datasets used and/or analyzed during the current study are available from the corresponding author on reasonable request.

## Abbreviations

CNS: Central nervous system
DAM: Disease-associated microglia
DEG: Differentially expressed genes
EAE: Experimental autoimmune encephalomyelitis
IFN: Interferon
LAM: Lupus-associated microglia
LPS: Lipopolysaccharide
ME/CFS: Myalgic encephalomyelitis/chronic fatigue syndrome
MGnD: Microglia with a neurodegenerative phenotype
NPSLE: Neuropsychiatric systemic lupus erythematosus
PET: Positron emission tomography
qRT-PCR: Quantitative real-time polymerase chain reaction
SLE: Systemic lupus erythematosus
TLR7: Toll-like receptor 7
