# Supplementary figures and images for: Unique primed status of microglia under the systemic autoimmune condition of lupus-prone mice
Source: Arthritis Res Ther. 2019 Dec 30;21:303. doi: 10.1186/s13075-019-2067-8 (PMC6936062; doi:10.1186/s13075-019-2067-8)

## Supplemental figure 1

## A Proteinuria

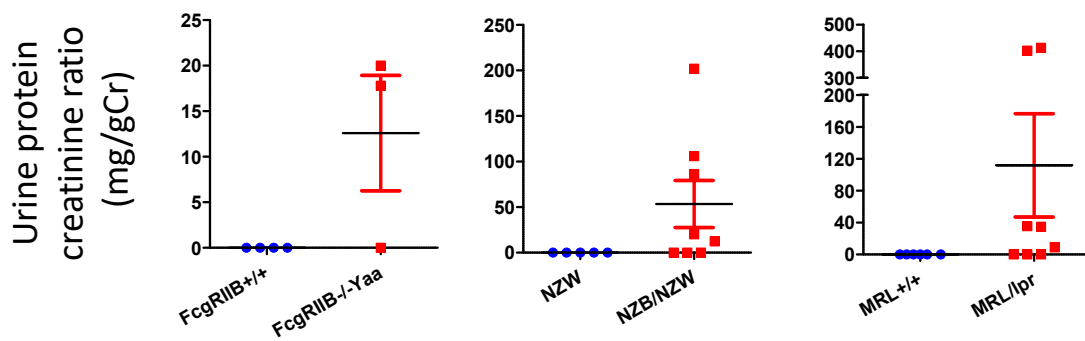

**B** Kidney PAS staining

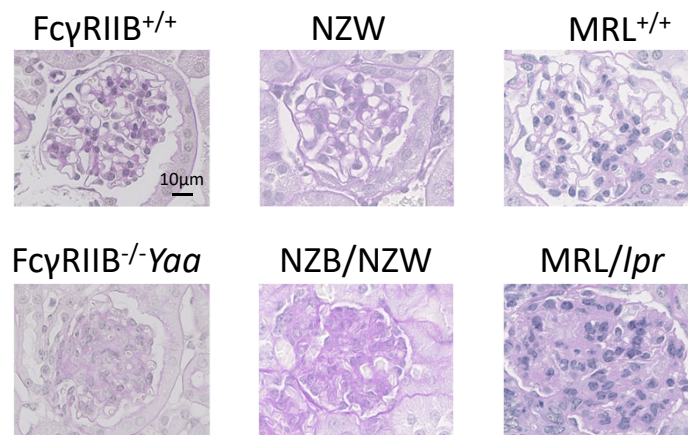

Supplement: Supplementary file 1 — Additional file 1: Figure S1. Nephritis was observed in three lupus models at the time of brain immunopathological analysis. (A) Proteinuria was observed in lupus models. (B) Representative images of PAS staining of the glomerulus in lupus model mice and their controls. [file 13075_2019_2067_MOESM1_ESM.pdf]

Additional file 2

Supplemental figure 2

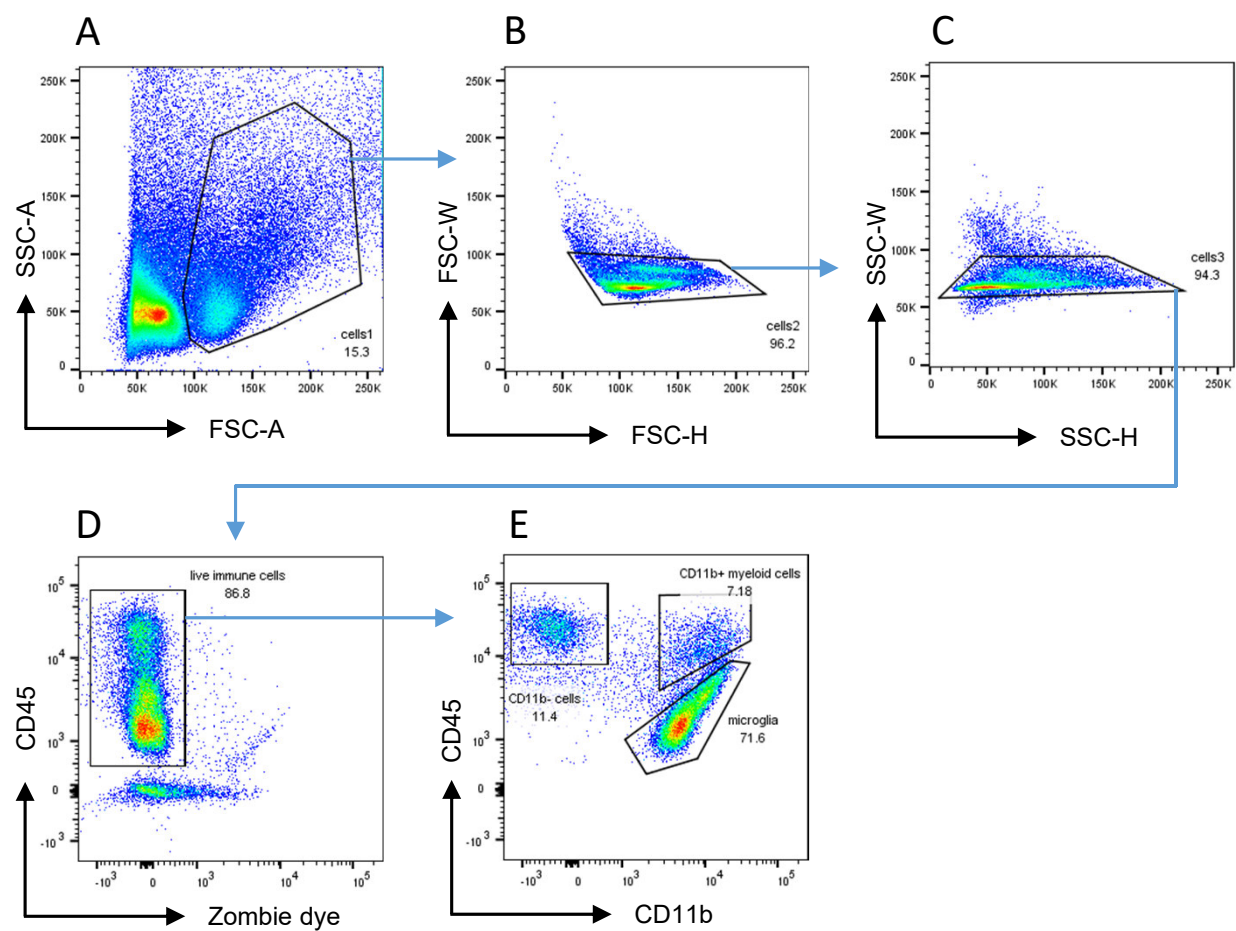

Supplement: Supplementary file 2 — Additional file 2: Figure S2. Gating strategy to analyze CD45+ cells from whole brain cells. Representative gating strategy for FcγRIIB-/-Yaa mice is shown. (A) Mononuclear cells were selected by size and granularity. (B, C) Doublet cells were excluded. (D) Live CD45+ cells were selected. (E) Populations shown in this plot were used for further analysis. [file 13075_2019_2067_MOESM2_ESM.pdf]

Additional file 3

Supplemental figure 3

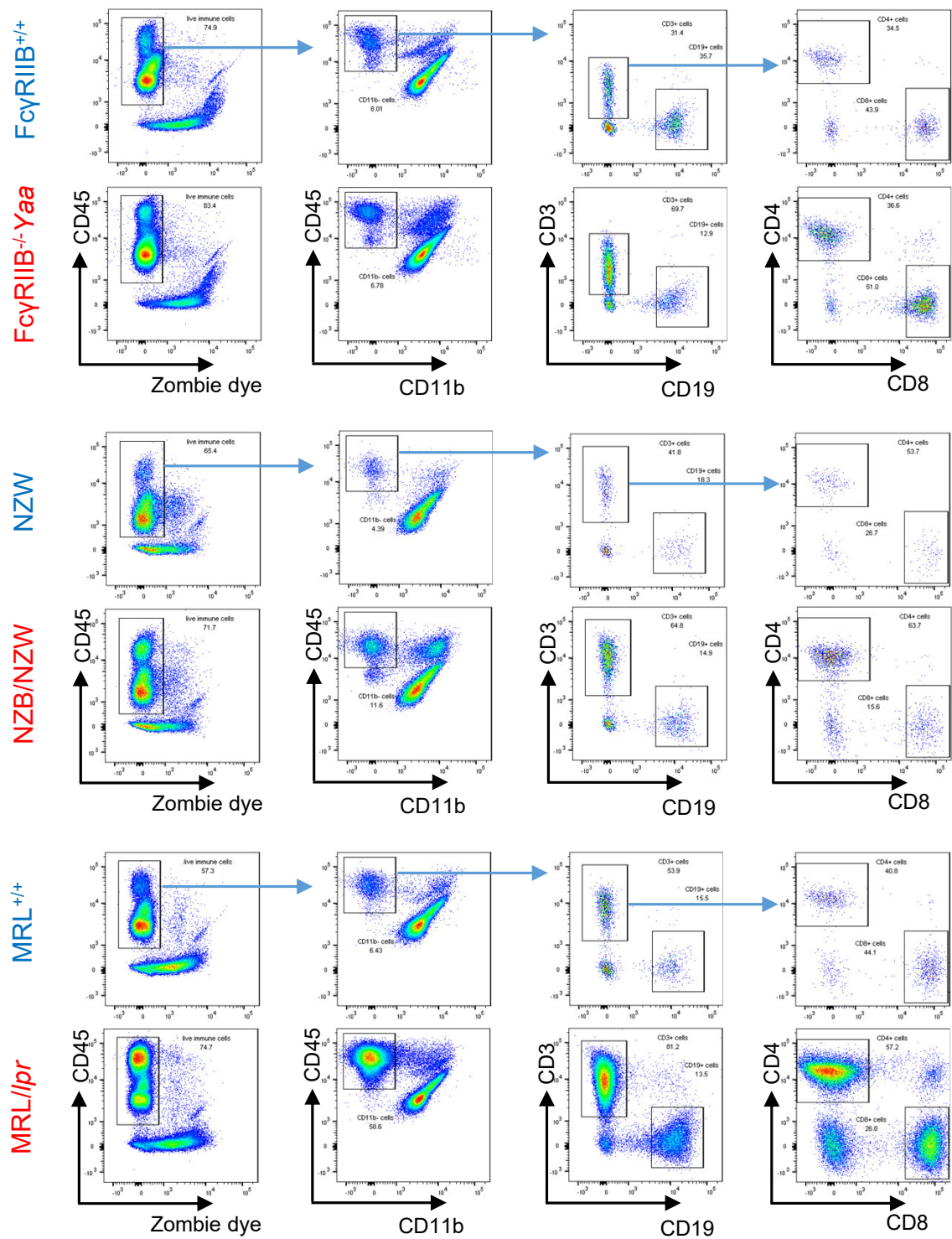

Supplement: Supplementary file 3 — Additional file 3: Figure S3. Gating strategy to analyze lymphocytes. Representative gating strategy for the analysis of three lupus models and controls is shown. [file 13075_2019_2067_MOESM3_ESM.pdf]

# Additional file 4

## Supplemental figure 4

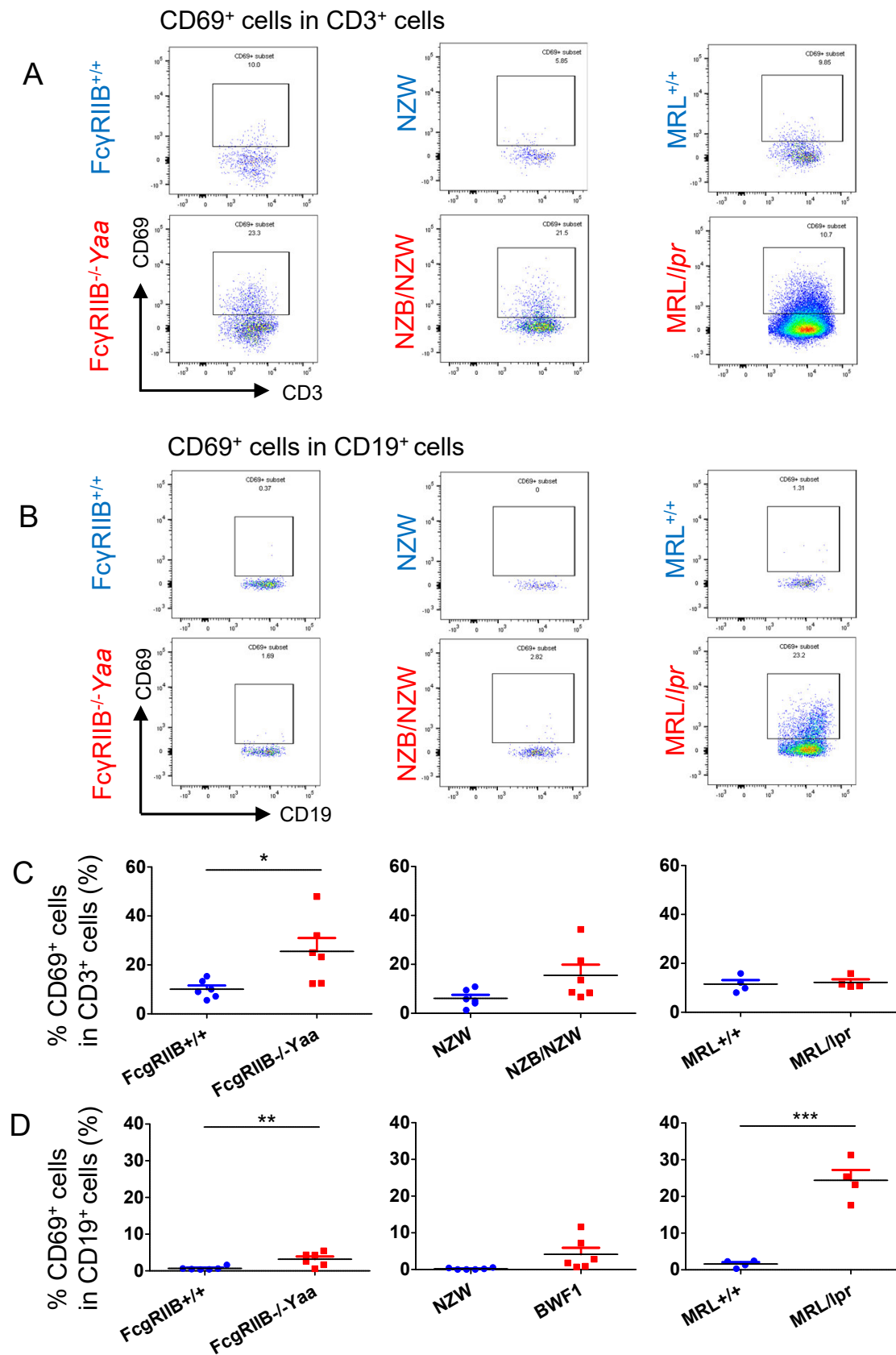

Supplement: Supplementary file 4 — Additional file 4: Figure S4. CD69 expression in lymphocytes from lupus prone mice. CD69 expression was analyzed by flow cytometry in three lupus prone mouse strains. (A) Representative plots of CD69 expression in CD3+ lymphocytes. (B) Representative plots of CD69 expression in CD19+ lymphocytes. (C, D) Comparison of CD69+ cells between lupus model mice and their controls. In C and D, symbols represent individual mice (n=6 for FcγRIIB-/-Yaa mice and controls, n=6 for NZB/NZW mice and controls, n=4 for MRL/lpr mice and controls) and horizontal lines indicate the mean and SEM. *P < 0.05, **P < 0.01, and ***P < 0.01 by Student’s t-test. [file 13075_2019_2067_MOESM4_ESM.pdf]

Additional file 6

Supplemental figure 5

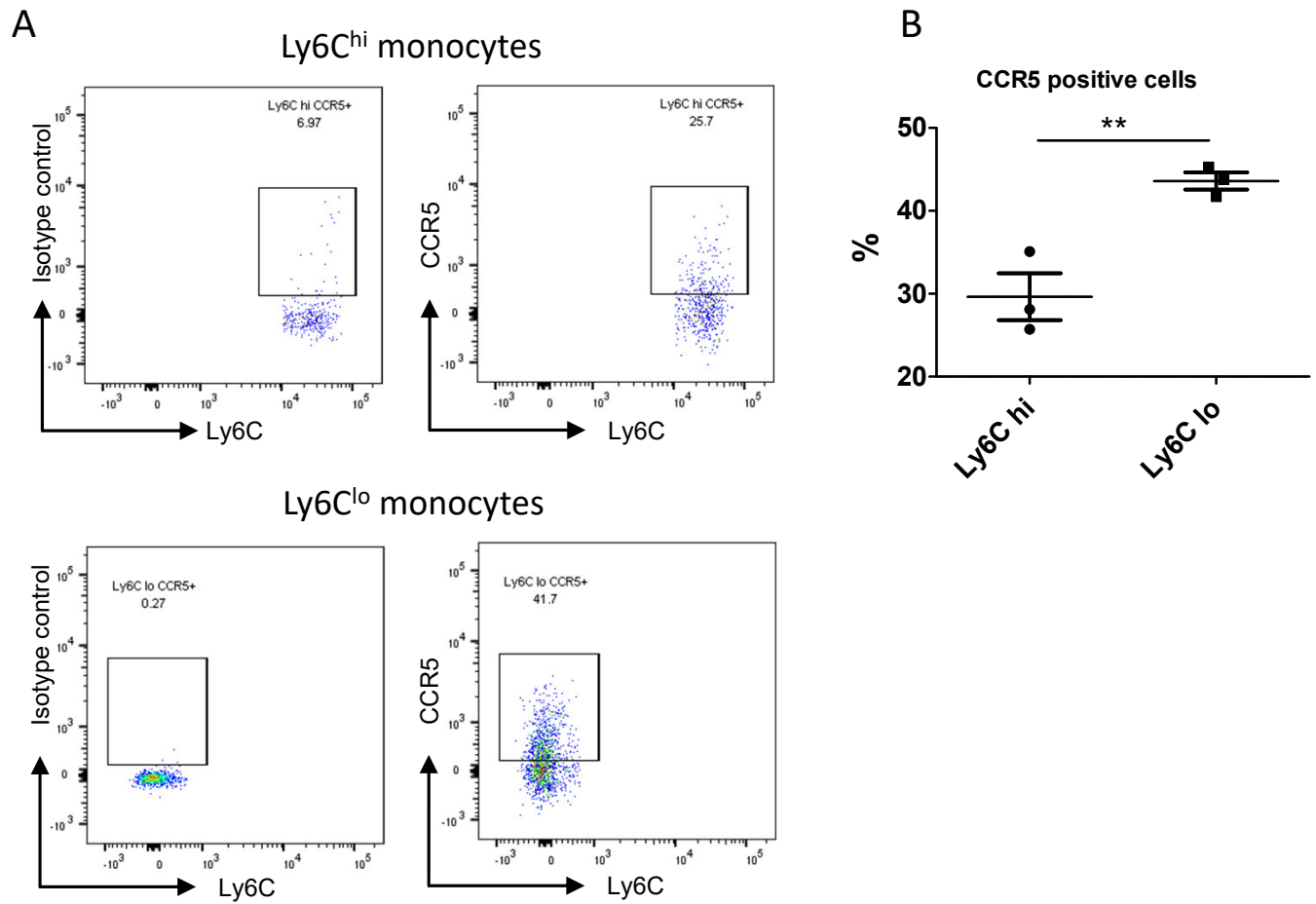

Supplement: Supplementary file 6 — Additional file 6: Figure S5. Expression of CCR5 in brain monocytes. Expression of CCR5 was examined by flow cytometry in FcγRIIB-/-Yaa mice. (A) Representative histograms of CCR5 expression in Ly6Chi monocytes and Ly6Clo monocytes. Staining of isotype controls is shown. (B) Comparison of CCR5 positive cells in Ly6Chi monocytes and Ly6Clo monocytes. Higher numbers of CCR5 positive cells were present in Ly6Clo monocytes compared with Ly6Chi monocytes. In B, Symbols represent individual mice (n=3) and horizontal lines indicate the mean and SEM. *P < 0.05, by Student’s t-test. [file 13075_2019_2067_MOESM6_ESM.pdf]
