# Supplementary material for: Unique primed status of microglia under the systemic autoimmune condition of lupus-prone mice
Source: Arthritis Res Ther. 2019 Dec 30;21:303. doi: 10.1186/s13075-019-2067-8 (PMC6936062; doi:10.1186/s13075-019-2067-8)
Supplement: Supplementary file 5 — Additional file 5: Table S1. Expressions of PDCA1 and MHC class I genes on RNA-seq analysis of microglia in FcγRIIB-/-Yaa mice and their controls. [file 13075_2019_2067_MOESM5_ESM.pdf]

Additional file 5

Supplemental table 1

Gene	Gene expression (FPKM)		$Fc\gamma RIIB^{-/-}$ *Yaa*/$Fc\gamma RIIB^{+/+}$	p-value	q-value
	$Fc\gamma RIIB^{+/+}$ (95% CI)	$Fc\gamma RIIB^{-/-}$ *Yaa* (95% CI)			
Bst2 (PDCA1)	30.3 (18.8, 41.7)	55.6 (37.9, 73.3)	1.83	< 0.00005	0.0049857
H2-K1	259.5 (186.2, 332.8)	492.9 (336.5, 649.3)	1.89	< 0.00005	0.0049857
H2-D1	151.1 (109.1, 193.1)	298.9 (210.2, 387.5)	1.97	< 0.00005	0.0049857
